# Midpalatal suture split after differential and fan-type expansion: a secondary analysis from a randomized clinical trial

**DOI:** 10.1590/2177-6709.30.6.e2525156.oar

**Published:** 2026-02-09

**Authors:** Daniela GARIB, Rodrigo TEIXEIRA, Rafaela Lopes CHACON, Ana Lúcia Alvares CAPELOZZA, José Roberto Pereira LAURIS, Camila MASSARO

**Affiliations:** 1 University of São Paulo, Bauru School of Dentistry and Hospital for Rehabilitation of Craniofacial Anomalies, Department of Orthodontics (Bauru/SP, Brazil).; 2 University of São Paulo, School of Dentistry, Department of Orthodontics (Bauru/SP, Brazil).; 3 University of São Paulo, School of Dentistry, Department of Radiology (Bauru/SP, Brazil).; 4 University of São Paulo, School of Dentistry, Department of Public Health (Bauru/SP, Brazil).

**Keywords:** Palatal expansion, Midpalatal suture, Cone beam computed tomography, Expansão palatina, Sutura palatina mediana, Tomografia computadorizada de feixe cônico

## Abstract

**Objective:**

To compare the opening of the midpalatal suture after rapid maxillary expansion (RME) with the expander with differential opening and the fan-type expander.

**Methods:**

Rapid maxillary expansion was performed with the expander with differential opening (n=12, 8 female, 4 male, mean age of 7.75 years) and the fan-type expander (n=12, 8 female, 4 male, mean age 8.08 years). CBCT scans were acquired before (T1) and immediately after (T2) the expansion. The outcomes comprised the opening of the midpalatal suture in the anterior and posterior nasal spine. Intergroup comparisons were performed with t or Mann-Whitney U tests (P<0.05).

**Results:**

All patients showed a midpalatal suture opening with greater increases in the anterior nasal spine compared to the region of the posterior nasal spine. The split in the anterior nasal spine was similar in both groups (P=0.153), while the opening in the posterior nasal spine was 1 mm greater in the differential expansion group (P<0.001). The greater increase in the anterior region of the midpalatal suture in both groups suggested a trapezoidal shape split.

**Conclusions:**

The expander with differential opening and the fan-type expander were capable of splitting the midpalatal suture in the mixed dentition. Despite both appliances showed similar opening in the anterior nasal spine, a greater expansion in the posterior region of the midpalatal suture was observed with the expander with differential opening.

## RESUMO

**Objetivo:**

Comparar a abertura da sutura palatina mediana após a expansão rápida da maxila realizada com o expansor maxilar com abertura diferencial e com o expansor em leque.

**Métodos:**

Expansão rápida da maxila foi realizada com o expansor maxilar com abertura diferencial (n=12; 8 do sexo feminino, 4 do masculino; média de idade de 7,75 anos) e com o expansor em leque (n=12; 8 do sexo feminino, 4 do sexo masculino; média de idade de 8,08 anos). Tomografias computadorizadas de feixe cônico foram obtidas antes (T1) e imediatamente após (T2) a expansão, para avaliação da abertura da sutura palatina mediana nas regiões das espinhas nasais anterior e posterior. As comparações intergrupos foram realizadas pelos testes t ou Mann-Whitney U (P<0,05).

**Resultados:**

Todos os pacientes apresentaram abertura da sutura palatina mediana, com maiores aumentos na região da espinha nasal anterior, em comparação ao efeito observado na região da espinha nasal posterior. A abertura anterior foi semelhante entre os grupos (P=0,153), enquanto a abertura posterior foi 1 mm maior no grupo tratado com expansão diferencial (P<0,001). O maior aumento na região anterior da sutura palatina mediana em ambos os grupos sugeriu uma geometria de abertura em formato trapezoidal.

**Conclusões:**

O expansor maxilar com abertura diferencial e o expansor em leque foram capazes de promover a abertura da sutura palatina mediana na dentição mista. Embora ambos os aparelhos tenham apresentado alterações semelhantes na região da espinha nasal anterior, observou-se maior expansão na região posterior da sutura palatina mediana com o expansor diferencial.

## INTRODUCTION

Rapid maxillary expansion (RME) has been recognized as an effective procedure when a transverse maxillary constriction is diagnosed.^1^ ^-^ ^5^ Maxillary base widening is achieved during expansion by the separation of the midpalatal suture and the circummaxillary sutural complex.^1^ ^,^ ^4^ ^-^ ^6^ A variety of maxillary expander designs have been successfully presented for this purpose, including Haas-type, Hyrax, fan-type, differential opening and bonded expanders.^1^ ^,^ ^7^ ^-^ ^15^ Regardless of the expander design, RME proved to be an effective procedure to produce transverse skeletal effects in the maxilla by means of an orthopedic effect.

The clinical evidence of midpalatal suture separation after RME is the gradual opening of a diastema between the maxillary central incisors.^1^ ^,^ ^5^ The split of the midpalatal suture can be easily confirmed in an occlusal radiography of the maxilla, acquired immediately after the active phase of the expansion.^4^ ^,^ ^5^ ^,^ ^16^ However, occlusal radiographs have a limitation in the posterior area of the midpalatal suture, attributed to the overlapping of the cranial base structures.^5^ ^,^ ^16^ ^-^ ^18^ Evaluation of a mixed-dentition dried skull after RME demonstrated a nonparallel opening at the midpalatal area with a wide opening at anterior nasal spine (ANS) and a gradual narrowing toward the posterior nasal spine (PNS).^5^ Tomographic assessment of the midpalatal region in deciduous and mixed dentitions confirmed that the PNS undergoes impact of the RME.^16^ ^,^ ^19^

Computed tomography (CT) allows quantitative evaluation of the bone changes in three dimensions without image superimposition.^17^ ^,^ ^18^ A previous CT study showed that the average opening of the midpalatal suture was 2.2 mm in the ANS and 0.9 mm in the PNS using a Haas-type expander in patients in the deciduous and mixed dentitions.^16^ Another CT study found that the amount of expansion in the PNS was approximately 40% of that observed in the anterior region of the midpalatal suture.^19^ A previous systematic review showed midpalatal suture openings of 12-52% of the total screw activation in young patients.^18^ Regarding the morphological aspects of suture opening, both parallel and triangular shapes were previously described after conventional RME.^5^ ^,^ ^16^ ^-^ ^20^ When a triangular shape was reported, the largest opening was at the anterior region.^5^ ^,^ ^16^ ^,^ ^19^ ^,^ ^20^

The expander design used for RME may influence the pattern of midpalatal suture split. Previous studies evaluated the opening of the midpalatal suture split using conventional expanders that open parallelly as the Haas and Hyrax-type expanders.^5^ ^,^ ^6^ ^,^ ^16^ ^,^ ^19^ ^-^ ^22^ The expander with differential opening (EDO) and the fan-type expander (FE) produced greater expansion in the anterior than the posterior region of the maxillary dental arch.^10^ ^-^ ^13^ These two expanders have in common an anterior screw. However, the EDO has a second screw posteriorly positioned while the FE has only a posterior hinge.^7^ ^,^ ^8^ Compared to the FE, the EDO showed greater transverse expansion at the level of orbital, zygomatic bone, nasal cavity, palatine foramen and molars.^10^ ^,^ ^14^ A previous comparison between the Hyrax expander and the EDO using occlusal radiographs, demonstrated a greater opening of the anterior region of the midpalatal suture in the EDO group compared to the conventional group.^13^ Conversely, a similar pattern of the midpalatal suture splitting was observed following expansion with both the Hyrax and differential expanders. Both EDO and Hyrax produced a slightly greater suture opening compared to the Haas-type expander; however, the differences did not exceed 1 mm.^22^ Midpalatal suture split produced by fan-type expansion was not previously described in the literature. Additionally, no previous study assessed the effects of the EDO and the FE in the posterior region of the midpalatal suture. Do the differences in the appliance design and the presence of a second screw influence the pattern of the midpalatal suture split in the mixed dentition?

The aim of the present study was to compare the amount and shape of the midpalatal suture split after RME with the expander with differential opening and the fan-type expander. The null hypothesis is that both expanders produce similar midpalatal suture opening after RME in the mixed dentition. 

## METHODOLOGY

### TRIAL DESIGN

This study was a secondary data analysis from a previous single-center randomized clinical trial (RCT) with two-parallel arms and a 1:1 allocation ratio (Bauru School of Dentistry, University of São Paulo, Bauru/SP, Brazil).^10^ ^,^ ^14^ The Consolidated Standards of Reporting Trials guidelines (CONSORT) were followed. Before trial commencement, the study was approved by the Research Ethics Committee of the Bauru School of Dentistry, and written informed consents were obtained from all patients and parents or legal guardians. 

Sample size calculation was based on a preliminary statistic with 5 patients from each group. For a standard deviation of 0.7 mm for the midpalatal suture opening in the anterior nasal spine, and a minimal intergroup difference of 1 mm to be detected, a sample of 8 subjects in each group was required to provide a statistical power of 80% with an alpha of 5%.

### PARTICIPANTS

All patients were recruited and treated by the same orthodontist at the Orthodontic Clinic of the Bauru School of Dentistry. The inclusion criteria comprised individuals of both sexes from 7 to 11 years of age with maxillary constriction and posterior crossbites. Exclusion criteria included individuals with Class III malocclusion, craniofacial syndromes, clinical absence of maxillary deciduous canines, and history of previous orthodontic treatment.

Randomization was performed using the website randomization.com (http://www.randomization.com).

Opaque, sealed, and sequentially numbered envelopes were prepared and opened for each patient following the sequence generated by the randomization.

Blinding of both patient and orthodontist was not possible in this study due to the differences in the appliance designs. The outcome assessment was not blind because the CBCT scans were acquired with the expander in the oral cavity. The generation of randomization list, allocation concealment, and implementation were performed independently by different researches.

### INTERVENTIONS

**Figure 1:**
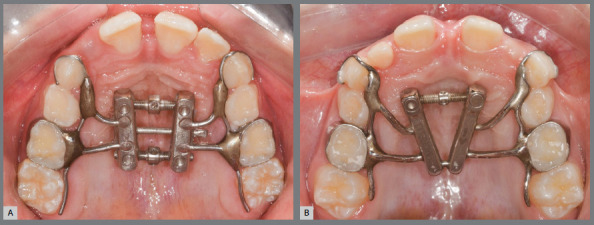
A) Maxillary expander with differential opening immediately after the active phase of rapid maxillary expansion. B) Fan-type expander immediately after the active phase of rapid maxillary expansion.

Patients were randomly allocated into two study groups. In the EDO group, the expander with differential opening (Peclab Ltda., Belo Horizonte/MG, Brazil) (Fig. 1A) was used to treat 12 patients (8 female, 4 male; initial mean age of 7.75 years). In the FE group, the fan-type expander (Morelli Ortodontia, Sorocaba/SP, Brazil) (Fig. 1B) was used to treat 12 patients (8 female, 4 male; initial mean age of 8.08 years). All patients were in the mixed dentition. In both groups, orthodontic bands were adapted on the maxillary second deciduous molars and C shape clasps were bonded on the maxillary deciduous canines. Additionally, a 1.0-mm stainless steel wire extension was contoured along the palatal surface of the permanent first molars (Fig. 1). The active phase of the expansion comprised 10 consecutive days following a screw activation rate of 2/4 turns in the morning and 2/4 turns in the evening (0.8 mm per day) for both groups. In the EDO, the anterior and posterior screws were concurrently activated for 6 days. Afterwards, additional 4 days of activation was performed only in the anterior screw. An 8-mm expansion was reached both in the FE screw and in the EDO anterior screw. After the active phase, the expanders were maintained in the oral cavity for 6 months in both groups.

CBCT scans were obtained prior to (T1) and immediately after (T2) the active phase of the RME. CBCT images were analyzed using the Nemoscan software (Nemotec, Madrid, Spain). Before measurement, the head position was standardized using the palatal plane parallel to the horizontal plane in the sagittal view, the infraorbital plane parallel to the horizontal plane in the coronal view, and the vertical plane coincident with ANS and PNS in the axial view.

### OUTCOMES

The outcomes assessed in the present study were the distance between the right and left margins of the midpalatal suture at the level of the anterior and posterior nasal spines at T1 and T2. 

### STATISTICAL ANALYSIS

All measurements were performed by the same examiner. Half of the sample was measured twice after at least 30 days. Intra-examiner reliability of the measurements was assessed using intraclass correlation coefficients (ICC).

Baseline characteristics were compared using Chi-square or *t*-tests. Normal distribution was verified with Shapiro-Wilk test. Intergroup comparisons were performed with t or Mann-Whitney U tests. All statistical analyses were performed using Statistica software (Statistica for Windows version 11.0; StatSoft, Tulsa, Okla). The level of significance considered was 5%. 

## RESULTS

Comparisons at baseline were similar in both groups (Table 1). Rapid maxillary expansion was successfully performed in all patients in the EDO and FE groups leading to midpalatal suture split.

**Table 1:** Intergroup comparisons for initial age, sex ratio, intercanine and intermolar distances and presence of posterior crossbite.

Variable		EDO (n=12) Mean (SD)	FE (n=12) Mean (SD)	P
Initial age (years)		7.75 (1.13)	8.08 (1.16)	0.485 †
Sex	Male	4	4	1.000 §
	Female	8	8	
Intercanine distance (mm)		29.7 (1.88)	28.5 (2.61)	0.230 †
Intermolar distance (mm)		48.7 (2.67)	47.7 (3.24)	0.433 †
Posterior crossbite	Unilateral	9	7	0.386 §
	Bilateral	3	5	

EDO= expander with differential opening; FE= fan-type expander; SD= standard deviation; † t test; § chi-square test.

Table 2 shows the results for the error study. Intraclass correlation coefficient showed that reliability was excellent (0.993) and good (0.846) for measurements performed at the ANS and PNS, respectively.

**Table 2:** Error study assessed with Intraclass Correlation Coefficients (ICC).

Variables		First measurement	Second measurement	Difference	ICC
		Mean (SD)	Mean (SD)	Mean (SD)	
Midpalatal suture opening	Anterior region	3.95 (1.56)	3.99 (1.58)	-0.04 (0.19)	0.993
	Posterior region	1.41 (0.65)	1.35 (0.53)	0.06 (0.32)	0.846

SD= standard deviation.

Intergroup comparison is shown in Table 3. A similar opening at the level of ANS was observed for patients treated with the EDO and the FE (P=0.153). However, changes at the level of PNS were 1.1 mm greater in patients treated with the EDO compared to FE (P<0.001). 

**Table 3:** Intergroup comparisons for the midpalatal suture opening at the anterior (t test) and posterior (Mann-Whitney U test) regions.

Variables		EDO (n=12) Mean (SD)	FE (n=12) Mean (SD)	95% CI Lower; Upper	P
Midpalatal suture opening	Anterior region	4.57 (1.31)	3.78 (1.30)	-0.31; 1.90	0.153
	Posterior region	1.87 (0.56)	0.72 (0.37)	0.74; 1.55	<0.001*

EDO= expander with differential opening; FE= fan-type expander; SD= standard deviation; CI= confidence interval; * Statistically significant at P<0.05.

**Figure 2:**
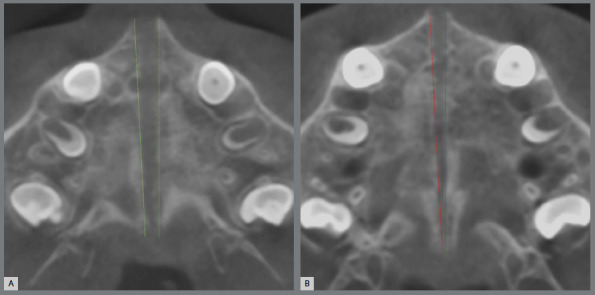
A) CBCT axial reconstruction immediately after rapid maxillary expansion of a patient treated with the expander with differential opening. B) CBCT axial reconstruction immediately after rapid maxillary expansion of a patient treated with the fan-type expander. The green and red lines illustrate the midpalatal sutural opening after expansion with EDO and FE, respectively.

Opening of the midpalatal suture after RME showed a trapezoidal shape in both groups with greater increases observed in the anterior, compared to the posterior region (Fig. 2).

## DISCUSSION

In the present study, the effects of two RME expanders indicated for a greater expansion in the anterior region of the dental arch were compared regarding the outcomes on the midpalatal suture. The sample of the study was obtained from a previous randomized clinical trial. Dental arch dimensions changes and maxillary dentoskeletal outcomes were previously reported.^10^ ^,^ ^14^ The same activation rate and endpoint was used for all patients (anterior screw opening of 8 mm in 10 days of activation) Additionally, both groups were similar regarding age and sex distribution (Table 1). The similar demographic characteristics at baseline, and the standardized amount of activation in the anterior region ensured sample homogeneity and a viable comparison between the two study groups. 

Computed tomography proved to be an useful tool to assess RME outcomes in all the three dimensions.^17^ Image superimposition in bidimensional images do not allow RME changes evaluation at the level of the PNS.^5^ ^,^ ^16^ The use of CBCT scans in the present study granted assessment of the midpalatal sutural opening complete extension. The adequate level of intra-examiner correlation reported in this study showed reliable measurements (Table 2).

Previous studies demonstrated midpalatal suture opening varying from 12%-52% of the total screw expansion after conventional RME.^17^ ^,^ ^18^ The present findings corroborate the previously reported rates, showing midpalatal suture opening around 47% and 57% for the anterior region, and 9% and 23% for the posterior region, using FE and EDO, respectively (Table 3). A previous study with metallic implants showed that orthopedic expansion at the level of basal bone was approximately 50% of the screw activation after conventional RME in the mixed dentition.^23^ In the present study, similar rate of orthopedic expansion was found for both appliances at the level of the ANS. Opening at the anterior limit of the midpalatal suture after conventional RME was 2.2 mm in a CT study with patients aged from 5 to 10 years,^16^ and approximately 3 mm in patients from 8-14 years of age.^19^ These differences were attributed to different amount of expansion in the studies. No previous comparison between EDO and FE regarding midpalatal splitting was found. In the mixed dentition, assessment of the midpalatal suture opening using maxillary occlusal radiographs showed anterior opening of 5.4 mm with the EDO and 3.1 mm with the conventional Hyrax expander.^13^ As midpalatal suture opening is proportional to the amount of screw activation, a greater anterior activation of the anterior screw in the EDO led to greater split of the midpalatal suture at the region of the anterior nasal spine. In the present study, similar outcomes were observed for the split at the anterior region of the midpalatal suture in both groups (Table 3). These outcomes were expected since the amount of activation was similar in the FE and in the anterior screw of the EDO (8 mm).

Compared to both EDO and Hyrax expander, the FE showed minor changes in the intermolar and maxillary transverse distances.^10^ ^-^ ^12^ ^,^ ^14^ One could assume that the FE would not cause sutural split at the PNS due to the presence of a posterior hinge that offers resistance to the expansion in the posterior region. However, both EDO and FE opened the suture in the PNS region (Table 3, Fig. 2). Previous CT studies after conventional RME in the deciduous and mixed dentitions showed mean openings of approximately 1 mm at the PNS.^16^ ^,^ ^19^ The present results showed that the EDO caused greater suture separation at the PNS compared to the FE, with a mean intergroup difference of 1.1mm. The explanation is the presence of a posterior screw in the EDO while the FE has only a limiting hinge in the posterior region. The posterior hinge in the FE not only limited the RME transverse effects in the molars and nasomaxillary complex, as previously demonstrated,^10^ ^,^ ^14^ but also influenced the midpalate suture split at the posterior region.

As expected, the changes in the PNS were smaller compared to the ANS opening, corroborating previous CT studies performed with conventional opening expanders.^16^ ^,^ ^19^ ^,^ ^20^ Regardless of the type of expander, resistance to midpalatal split is greater in the posterior region due to the sutures of the maxilla with neighboring facial and cranial bones. RME with the Haas-type expander showed that the opening of the midpalatal suture in the ANS was 1.2 mm greater than expansion in the PNS in patients in the mixed dentition from 5 to 8 years of age assessed using CT exams.^16^ When comparing the split of the midpalatal suture in the anterior and posterior regions using CBCT coronal views of patients treated with Haas-type and Hyrax expanders, the anterior suture opening was approximately 1 mm greater than the posterior suture opening.^20^ In our study, the amount of expansion in the PNS was approximately 40% and 20% of that observed in the ANS for the EDO and FE, respectively.

The CBCT assessment provided adequate visualization of the midpalatal suture opening in the anteroposterior direction. A previous CT study using a Haas-type expander suggested that the greater the expansion, the more evident the triangular shape of the midpalatal split.^16^ Conversely, parallel opening of the midpalatal suture was previously demonstrated after conventional RME; however, the evaluation did not extend to the PNS, and the most posterior measurement was performed at the level of the maxillary first molar.^22^ ^,^ ^24^ In this study, midpalatal split occurred with a trapezoidal shape in both groups (Fig. 2). However, the differential expansion between the anterior and posterior regions of the suture was greater in the FE group compared to the EDO group (Table 3, Fig. 2). The greater posterior opening of the suture observed with the EDO is probably associated with the more significant skeletal outcomes also observed with this expander when compared to the FE.^14^ The previous reported difference between both expanders in the maxillary expansion at the level of the zygomatic bone and palatine foramen were 0.98 and 1.2 mm, respectively.^14^ In addition, the evaluation of the nasomaxillary region also showed greater changes in the lower level of the nasal cavity in patients treated with the EDO compared to those treated with FE.^25^ Clinically, this skeletal changes could benefit individuals with breathing disorders, given an advantage to the EDO design for these patients.

The stability of RME outcomes has been reported to be associated with an appropriate retention protocols and the magnitude of skeletal and dental changes achieved with treatment.^26^ To minimize relapses, an overcorrection in the transverse aspect and the maintenance of the expander in position for 6 months should be followed.^6, 27^ No previous study evaluated the stability of the outcomes obtained with EDO and FE. However, we may expect that more stable results can be accomplished when greater skeletal changes are achieved. In addition, a significant percentage of the skeletal changes tend to be maintained while higher relapse rates is observed in dental arch widths.^28^

A slight pain during screw activations is expected during RME procedure. In the present sample, 66% of the patients treated with the EDO reported some discomfort during the active phase of the expansion. In the fan-type group, this was reported by 50% of the patients. Parents or legal guardians did not report further difficulties during the activation of the screws. However, it is essential to emphasize the importance of providing clear instructions and demonstrating the activation procedure, while also addressing potential discomfort during treatment, to ensure parents feel confident during the active phase of expansion.

The EDO and the FE expanders were capable to open the anterior and posterior regions of the midpalatal suture in the mixed dentition with greater split at the ANS, creating a trapezoidal opening shape (Table 3, Fig. 2). Changes in the PNS were mild, especially in the FE group. Additional caution is recommended when evaluating the midpalatal suture split at the level of the PNS, considering that the voxel size used in this study (0.3 mm) has an average spatial resolution of 0.7 mm.^29^ Therefore, a limitation of this study is that an opening of the midpalatal suture smaller than 0.7 mm at PNS may be not identified using this methodology. Moreover, the generalizability of the present results is limited to the age range and sex distribution of the study sample, comprising patients in the mixed dentition of both male and female sexes. Finally, future studies could include a conventional expander group, treated with Haas-type or Hyrax expanders, for a further understanding of the influence of the expander design in the midpalatal suture behavior.

## CONCLUSIONS

» The expander with differential opening and the fan-type expander were effective in producing a split of the midpalatal suture, after expansion.
» In both groups, a trapezoidal opening split shape was observed in the midpalatal suture, with greater opening in the anterior nasal spine compared to the posterior nasal spine.
» The expander with differential opening produced a significantly greater posterior nasal spine opening compared to the fan-type. On the other hand, the sutural split at the level of the anterior nasal spine was similar for both expanders.
